# Maternal Health Service Uptake Is Associated with a Higher Skin-to-Skin Care Practice in Ethiopia: Result from a National Survey

**DOI:** 10.1155/2020/8841349

**Published:** 2020-12-16

**Authors:** Dabere Nigatu, Gedefaw Abeje, Alemayehu G. Mekonnen, Muluken Azage, Daniel Bogale

**Affiliations:** ^1^Department of Reproductive Health and Population Studies, School of Public Health, College of Medicine and Health Sciences, Bahir Dar University, Bahir Dar, Ethiopia; ^2^Department of Nursing, College of Health Science, Debre Berhan University, Debre Berhan, Ethiopia; ^3^Department of Environmental Health, School of Public Health, College of Medicine and Health Sciences, Bahir Dar University, Bahir Dar, Ethiopia; ^4^Department of Public Health, College of Health Sciences, Arsi University, Asella, Ethiopia

## Abstract

**Background:**

Though skin-to-skin care (SSC) is becoming an important newborn care package at both facility and community levels in Ethiopia, there is a lack of evidence to monitor the progress at each level. Therefore, this study is aimed at quantifying the proportion of SSC at both national and regional levels and identifying factors that affect SSC uptake in Ethiopia.

**Method:**

We used the 2016 Ethiopia Demographic and Health Survey data. The survey employed a multistage cluster sampling method. We included 7,488 live births in the analysis. The factors influencing SSC practice were identified using a multivariable logistic regression model. We reported adjusted odds ratios (AORs) with 95% confidence intervals (CIs).

**Results:**

In Ethiopia, 24.3% of mothers practiced SSC for their newborns (95% CI = 23.3, 25.2). The highest proportion was in Addis Ababa (63%), and the lowest was in the Somali region (14.5%). Attending 1-4 antenatal care (AOR = 1.51, 95%CI = [1.08, 2.12], giving birth at health facility (AOR = 4.51, 95%CI = [2.16, 9.44], and having female births (AOR = 1.24, 95%CI = [1.01, 1.54]) were associated with more odds of practicing SSC. However, giving birth by the cesarean section had resulted in lower odds of practicing SSC (AOR = 0.37, 95%CI = [0.22, 0.63]). Regions with reduced odds of SSC practice include Amhara (AOR = 0.57, 95%CI = [0.40, 0.82]), Somali (AOR = 0.51, 95%CI = [0.31, 0.83]), and Southern Nations, Nationalities, and People (AOR = 0.64, 95%CI = [0.43, 0.94]).

**Conclusions:**

The SSC practice was low in Ethiopia with a high level of variation between regions. In Ethiopia, maternal health service uptake affects the SSC of the newborns. Well-tailored community-level interventions are needed to increase skin-to-skin care practice among home delivery mothers.

## 1. Background

Globally, the preterm birth rate was estimated to be 10.6%, equating to 14.8 million live preterm births in 2014. From these, 12 million (81.1%) of the preterm births occurred in Asia and sub-Saharan Africa [1]. In 2015, an estimated 20.5 million live births were low birth weight (LBW), 91% from low- and middle -income countries [2, 3]. The large burden of preterm births and low birth weight calls for more effective interventions for primary prevention including scale up of feasible and evidence-based interventions such as Kangaroo Mother Care (KMC) [4].

The KMC is care of preterm and/or low birth weight babies carried skin-to-skin with the mother including early breastfeeding and follow-up [5, 6]. Skin-to-skin care (SSC) is the prime component of KMC. The World Health Organization defined SSC as placing the naked baby on the mother's bare abdomen or chest immediately or in less than 10 minutes after birth or soon afterwards [7]. It is easy-to-practice method but has a substantial benefit in promoting the health and well-being of infants born preterm as well as full-term [5]. In resource-limited countries like Ethiopia, skin-to-skin care is an effective and safe alternative/substitute to conventional neonatal care for preterm/LBW infants [5, 8]. Previous studies revealed that SSC for preterm and/or low birth weight newborns associated with reduced mortality and morbidity [8–11], improved maternal-infant attachment (emotional and caring behavior) [8, 11–13], improved infant growth (increase weight, head circumference, and length gain) [8, 11, 14], and enhanced early initiation of breastfeeding [15]. However, SSC remains unavailable at scale in most low-income countries [10], and there is also a lack of studies about SSC in low-income countries [16].

In Ethiopia, SSC was introduced in 1996 at the Tikur Anbesa Hospital. Since then, the SSC services have been expanded to other hospitals and health facilities at all levels [17]. In settings like Ethiopia where many childbirths are taking place at home (up to 52%) [18], only facility-based implementation of SSC may not be adequate to bring the desired child health outcomes. In 2013, Ethiopia launched a community-based newborn care including SSC as a means of bringing life-saving care to mothers and newborns at the community level with Health Extension Workers and Women Development Armies as a vehicle of its implementation [19, 20]. Since then, integrated and multilevel interventions (i.e., both at community- and facility-level) were instigated in the most populous regions of Ethiopia (Amhara, Oromia, SNNP, and Tigray) to promote early SSC [21]. The community level early SSC promotion is irrespective of the birth weight. This is because of two main reasons: (1) there is no established community/household level system to ensure all births have measured birth weight in the country, and (2) more than half of the births are taking place at home. Even there are births occurred at a health facility, but lack registered birth weight. Regarding the succees story, there are evidence of improving newborn care at the community level and increased maternal satisfaction and empowerment with the care of their newborns [13, 20, 22]. Therefore, SSC becomes the high impact newborn care package at both facility and community levels in Ethiopia [23]. Thus, both national and/or subnational research are becoming the cornerstone of the strategy to monitor progress at the desired level. However, except for few pocket studies done so far, there is inadequate data on the proportion of SSC at the national level. Therefore, the current study is aimed at determining the proportion of SSC at both national and regional levels. Moreover, the study identified factors that affect SSC uptake using a nationally representative household survey. The SSC at birth is one of the strategic interventions in place in the country to reduce neonatal and child morbidity and mortality. Thus, the current findings can be applied to improve the level of skin-to-skin care practice at the community and health facility levels. It can also be used as an essential input to track or evaluate the performance of programs that have been in place in the area of skin-to-skin care practice in the country.

## 2. Methods

### 2.1. Data Sources

The data source for this analysis was the 2016 Ethiopia Demographic and Health Survey (EDHS). It is a nationally representative household survey carried out every five years since 2000. The datasets of EDHS surveys are freely available, and we have accessed it from the online repository of the Demographic and Health Survey (DHS) Program website upon request via a link https://www.dhsprogram.com/data/available-datasets.cfm.

### 2.2. Sample Size and Sampling Procedure

The sample for the 2016 EDHS was designed to provide estimates of key indicators both at national and regional levels. List of enumeration areas (EAs) generated for the 2007 Ethiopia Population and Housing Census was used as a sampling frame for the 2016 EDHS. The DHS used a stratified two-stage cluster sampling technique. In the first stage, 645 EAs (202 urban and 443 rural areas) were selected with probability proportional to the EA size. Then, household listing was done for the selected EAs. In the second stage, a fixed number of 28 households per cluster were selected using the newly created household list as a sampling frame [24].

**Figure 1:**
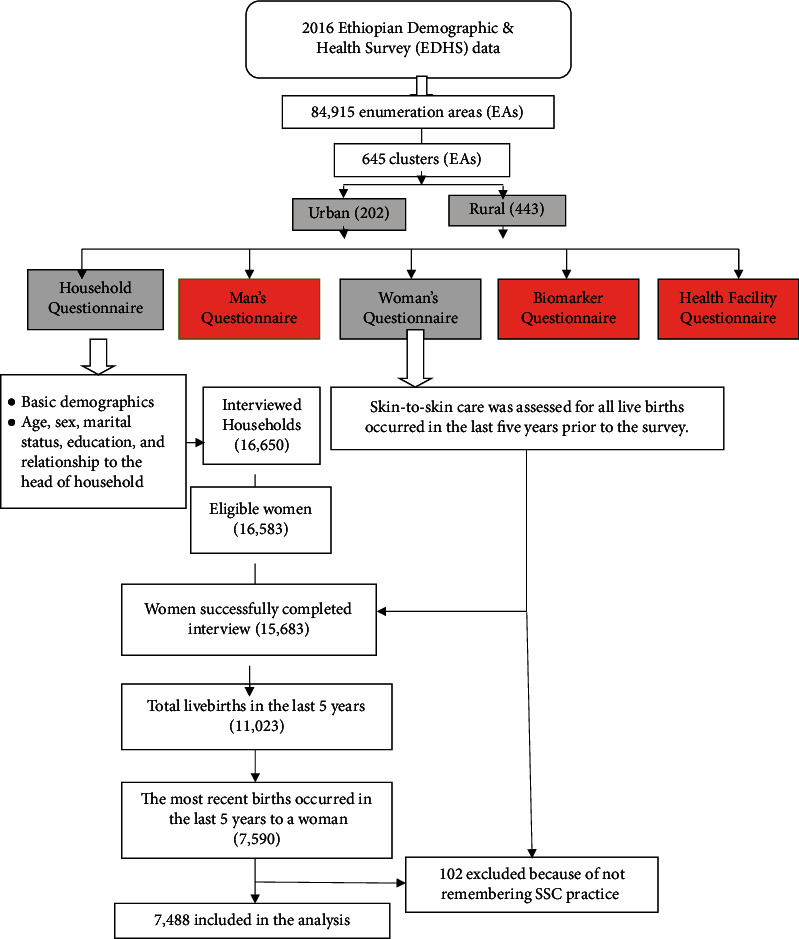
The sample selection procedure flowchart for skin-to-skin care practice, EDHS, 2016.

A total of 11,023 live births born from 15,683 women in the last 5 years preceding the survey. This study considered only the most recent births to a woman taking place in the five years preceding the survey. For two reasons, we preferred to rely only on the most recent births. The first reason is to reduce recall bias, and the second reason is that some important variables (for example, antenatal care) are assessed only for the most recent births. When we consider only the last birth to a woman, we reached a sample of 7,590 live births. From 7,590 live births, we excluded 102 mothers who did not remember SSC practice for their last birth. Therefore, the current analysis included 7,488 weighted sample (7,060 unweighted sample) of index live births (Figure 1).

### 2.3. Study Variable Measurement

Skin-to-skin care is an outcome variable. In the 2016 EDHS, mothers who gave live births in the last five years preceding the survey were asked about skin-to-skin care practices. The question was articulated this way: “immediately after the birth, was (NAME) put directly on the bare skin of your chest?” The response options were “yes”, “no,” or “I don't know.” We excluded the “I do not know” responses from the analysis.

The explanatory variables include maternal age at delivery, maternal education, maternal current employment status, maternal current marital status, place of residence, region, wealth index, antenatal care visits, place of delivery, cesarean section (CS) delivery, number of living children, sex of the child, baby's size at birth (as reported by mothers), parity, and childbirth attendant. The details of explanatory variables coding are provided as supplementary material (see Table S1). The 2016 EDHS has a question to assess maternal perceptions of the baby's birth size. The mothers were asked to retrospectively classify their babies' sizes at birth as “very large,” “larger than average,” “average,” “smaller than average,” or “very small.” Then, we recoded the birth size into two categories: very large, larger than average, and average responses taken into a category of “average or above average”, whereas smaller than average and very small responses taken into a category of “small.”

The DHS collected information about the number and kinds of consumer goods and the housing characteristics a household owns. These data were used to generate wealth index scores using principal component analysis. Finally, the wealth index scores were categorized into quintiles [24, 25].

### 2.4. Data Analysis

The data was analyzed using the STATA version 14.0 statistical software package. Recoding of some variables was done to suit the analysis. We considered sample weighting and sampling design during analysis because the EDHS data derived from a complex survey. We have used the SVYSET Stata command to declare a complex survey design for our dataset and the SVY command whenever we run estimates.

Multivariable logistic regression model was fitted using binary logistic regression analysis. Variables having *p* value less than 0.25 in the bivariable logistic regression analysis were candidate variables for the multivariable logistic regression model [26]. The enter method was applied to fit the logistic regression model. The association between explanatory variables and outcome variable (skin-to-skin care practice) was quantified using odds ratio with 95% confidence interval. The presence of significant association was declared at *p* value less than 0.05, or if the reported confidence intervals for odds ratio do not include one.

This manuscript is prepared following the Strengthening the Reporting of Observational Studies in Epidemiology (STROBE) checklist for cross-sectional study reporting guidelines [27] (Table S2).

### 2.5. Ethical Considerations

The National Research Ethics Review Committee at the Federal Democratic Republic of Ethiopia Ministry of Science and Technology and the Institutional Review Board of ICF International reviewed and approved the 2016 EDHS survey protocol. The authors got a permission from the ICF-DHS program to use the DHS data and accessed through “https://www.dhsprogram.com/data/dataset_admin/login_main.cfm.” Interested readers can refer the 2016 EDHS country report for further information about the survey protocol [24].

## 3. Results

### 3.1. Sociodemographic Characteristics and Maternal Health Services Uptake

**Figure 2:**
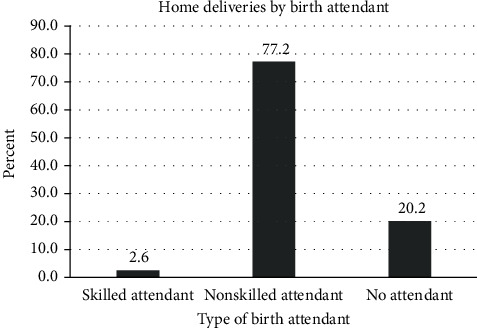
The distribution of home deliveries by type of birth attendants, EDHS 2016. Skilled attendant includes doctors/physicians, nurses, midwives, public health officers, and health extension workers; nonskilled attendant includes traditional birth attendants, relatives/friends/neighbors, and other persons.

A total of 7,488 mothers who had live births were included in the present analysis. A sample of 7,488 was achieved after exclusion of 102 mothers who did not remember skin-to-skin care practice for their last birth. Eighty percent of the mothers were in the age range of 19-34 years. About two-thirds, 63.5%, of mothers had no formal education. About 88% of the mothers were rural residents, and seven out of ten mothers were not employed. Majority, 94%, of the mothers were married. About 19% of the mothers were at their first birth, and 27.2% of the babies were small size at birth. Thirty-seven percent of the mothers did not attend antenatal care for their index pregnancy, 69% gave birth at home, 32.4% assisted by skilled birth attendant, and 1.8% gave birth by the cesarean section (Table 1). From 5171 home deliveries, 77.2% assisted by nonskilled birth attendants, and 20.2% delivered without any assistant (Figure 2). The distribution of the birth assistant by regions in Ethiopia and type of residence (urban/rural) was provided as supplementary data (Table S3).

**Figure 3:**
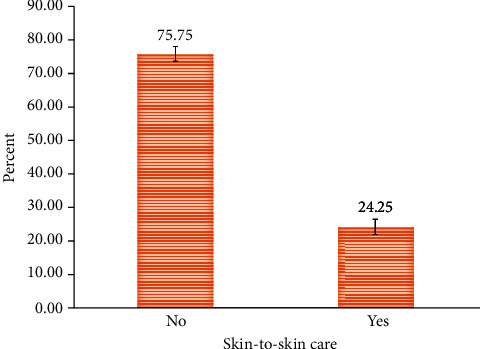
The proportion of mothers who had practiced skin-to-skin care in Ethiopia, EDHS, 2016.

In Ethiopia, 24.3% of mothers practiced skin-to-skin care for their newborns (95%CI = 23.3, 25.2) (Figure 3). The proportions of skin-to-skin care varies across regions of Ethiopia; the highest was in Addis Ababa (63%), and the lowest was in the Somali region (14.5%). The proportion of skin-to-skin care practices was higher among urban mothers (53%) compared to rural mothers (20%). The proportion of SSC was as low as 10% among mothers who had no antenatal care visit during the index pregnancy and as high as 41% among mothers who had attended more than four ANC visits during the index pregnancy. About 59% of the mothers who gave birth at health facilities practiced SSC, while only 9% of the mothers who gave birth at home practiced SSC for their most recent birth. Higher proportion of mothers practiced SSC if they were primiparous (35.6%) than multiparous (21.6%). The proportion of SSC practice was higher for mothers who gave birth by CS (35.6%) as compared to mothers who gave birth without CS (24%). Higher proportion of mothers who were assisted by skilled birth attendant (57%) practiced SSC as compared to mothers who were assisted by nonskilled attendant (8%) (Table 1).

### 3.2. Factors Affecting Skin-to-Skin Care Practice

This study revealed the presence of significant differences in SSC practice by residential regions, ANC status, place of delivery, mode of delivery, and sex of the child. As compared to mothers who lived in the Tigray region, those mothers who lived in the Amhara region (AOR = 0.57, 95%CI = [0.40, 0.82]), Somali region (AOR = 0.51, 95%CI = [0.31, 0.83]), and SNNP region (AOR = 0.64, 95%CI = [0.43, 0.94]) had lower odds of SSC practice (Table 2).

We found that attending ANC, giving birth at health facility, and mode of delivery had an effect on SSC practice. Mothers who had attended 1-4 ANC (AOR = 1.51, 95%CI = [1.08, 2.12] visits had higher odds of SSC practice compared to those mothers who had no ANC visit. Similarly, those mothers who gave birth at health facilities were about 2 times higher odds of SSC practice (AOR = 4.51, 95%CI = [2.16, 9.44]) than those mothers who gave birth at home. However, those mothers who gave birth by CS had 63% less odds of practicing SSC (AOR = 0.37, 95%CI = [0.22, 0.63]). The odds of practicing SSC were higher if a child was female than male (AOR = 1.24, 95%CI = [1.01, 1.54]) (Table 2).

## 4. Discussion

The current study showed the presence of large variations in skin-to-skin care practice between regions of Ethiopia. It also revealed the role of ANC follow-up, place of delivery, mode of delivery, and sex of children for skin-to-skin care of newborns immediately after birth in Ethiopia.

The national level proportion of SSC practice was 24.3% (95% CI: 23.3, 25.2) with substantial variation between regions of Ethiopia. Addis Ababa (63%) was the region with the highest proportion of skin-to-skin care, while the Somali region (14.5%) was with the lowest proportion of skin-to-skin care. The high variation among regions might be because of the gap in the initiation and scale up of the community-based newborn care practices across the regions. Usually, most maternal, newborn, and child health programs including community-based newborn care packages are piloted/introduced in the four most populous regions of Ethiopia—Amhara, Oromia, Tigray, and the Southern Nations, Nationalities, and People's (SNNP) regions. For example, the last ten kilometers (L10K) project [28, 29], the IDEAS (Informed Decisions for Actions in Maternal and Newborn Health) project [19], and MCHIP (Maternal and Child Health Integrated Program) [21] were initiated and implemented in these four regions. The national proportion is greater than the proportion in India (14.5%) [30] and in Nepal (16.5%) [31] where as it is lower than the proportion reported in Sri Lanka (50.4%). The Sri Lanka finding is higher than our finding because the Sri Lanka study is a preintervention survey among deliveries in the health facility. A multisite study reported SSC practice in Eastern India (15%) and in Bangladesh (30%) [32].

In this study, attending ANC had positively influenced SSC of newborns during the immediate postpartum period. This might be due to the influential role of antenatal counseling on immediate newborn care practices including skin-to-skin care of newborns. Existing evidence also supports the positive association between receiving antenatal counseling and SSC practice during the immediate postpartum period [21]. Besides, a qualitative study remarked that antenatal awareness could be a feasible remedy to overcoming knowledge and attitude related-barriers of KMC practice in the community [33].

The current study also suggested that giving birth at a health facility significantly increased skin-to-skin care practice of newborns. In fact, giving birth at a health facility can give a better opportunity for health professionals to assess newborn conditions and suggest mothers for skin-to-skin care of their newborns. Another study done in Ethiopia also reported that SSC was significantly higher among facility births than home births [21].

We observed a higher proportion of SSC practice among mothers who gave birth by CS than without CS. When the role of confounders controlled via the multivariable regression analysis, we found reversed association. Giving birth via the cesarean section had a negative influence on practice of skin-to-skin care. This might be because of the fact that those women who undergo CS may take longer time to recover from anesthesia, and the CS site may be painful and need time to heal. These problems combined make positioning the newborn in skin-to-skin contact with the mother difficult during the immediate postpartum period. A systematic review of evidences from low- and middle-income countries also reported pain/fatigue as top-ranked barriers of KMC practice [34].

The sex of the newborn also affects skin-to-skin care practice of the mothers. In our findings, mothers have a greater tendency to practice skin-to-skin care for female newborns. This finding may be difficult to explain, but it may be associated because of the birth size/weight difference between the two sexes. The birth size/weight differences between the two sexes evidenced in several studies reported that male newborns tended to be larger and heavier than females at birth [35–37].

The current study had evaluated the relationship between the baby's birth size and SSC practice. The maternally perceived baby size at birth did not show significant association with skin-to-skin care practice of newborns during the immediate postpartum period. The lack of association might be because of inaccurate classification of the baby size at birth. The problem of misclassification was addressed in a previous study reporting that maternal assessment of the baby size is an inaccurate proxy indicator of low birth weight in Ethiopia [38]. This implies the importance of noncriteria-based (i.e., irrespective of birth size) application of SSC at the community level.

Being a nationally representative data with large sample and making use of the most recent births taking place in the last five years prior to the survey can be the strength of this study. However, the results of this study may not be free of recall bias because skin-to-skin care practice was assessed for the index live births taking place in the last five years preceding the survey. Birth weight, a potential predictor variable for SSC practice, was not considered because of a large missing (85%) in the 2016 EDHS data [24, 38]. We have considered the baby's birth size which is a proxy indicator of birth weight where there is a lack of data on birth weight [39].

## 5. Conclusions

The proportion of skin-to-skin care practices was low in Ethiopia with significant variation across regions. The study also revealed the significant role of ANC follow-up, place of delivery, mode of delivery, and sex of child for SSC of newborns immediately after birth in Ethiopia. We recommend a community-based promotion of SSC at full scale to increase skin-to-skin care practice and minimize the existing regional variations in SSC practice in the country. Additionally, taking measures to increase ANC uptake and strengthening ANC counseling with due consideration of skin-to-skin care component can enhance the SSC practice in the community.

## Figures and Tables

**Table 1:** Proportion of SSC by sociodemographic characteristics and maternal health services in Ethiopia, EDHS 2016.

Variables	Frequency	Percent	Proportion of SSC practice with 95% CI
Maternal age at delivery			
< =18	558	7.5	24.3 [19.25,30.20]
19-34	5,612	74.9	25.8 [23.39,28.31]
> =35	1,318	17.6	17.7 [14.38,21.68]
Educational status			
No formal education	4,753	63.5	18.1 [15.75,20.71]
Primary	2,118	28.3	29.8 [26.68,33.12]
Secondary	412	5.5	54.0 [46.05,61.68]
Higher	205	2.7	49.9 [37.95,61.87]
Place of residence			
Urban	921	12.3	53.2 [46.81,59.47]
Rural	6,567	87.7	20.2 [17.88,22.72]
Regional distribution			
Tigray	529	7.1	47.8 [40.15,55.47]
Afar	69	0.9	15.8 [12.20,20.25]
Amhara	1,610	21.5	20.8 [17.58,24.36]
Oromia	3,099	41.4	20.6 [16.72,25.10]
Somali	266	3.6	14.5 [10.82,19.25]
Benishangul-Gumuz	80	1.1	30.3 [22.62,39.16]
SNNPR	1,585	21.2	25.6 [19.36,28.43]
Gambela	20	0.3	34.2 [28.18,40.67]
Harari	17	0.2	43.1 [35.74,50.69]
Addis Ababa	180	2.4	62.7 [55.80,69.12]
Dire Dawa	33	0.4	47.9 [40.92,54.98]
Wealth index			
Poorest	1,642	21.9	13.0 [10.12,16.60]
Poorer	1,642	21.9	19.0 [15.69,22.87]
Middle	1,574	21.0	22.2 [18.39,26.50]
Richer	1,411	18.9	26.2 [22.46,30.32]
Richest	1,219	16.3	46.8 [42.40,51.31]
Current employment status			
Not employed	5,353	71.5	22.2 [19.87,24.81]
Employed	2,135	28.5	29.3 [25.96,32.86]
Current marital status			
Never in union	56	0.75	39.3 [22.26,59.35]
Currently in union	7,011	93.6	24.2 [21.91,26.56]
Formerly in union	421	5.6	23.8 [18.88,29.61]
Sex of child			
Male	3,894	52.0	22.9 [20.43,25.53]
Female	3,594	48.0	25.7 [22.88,28.83]
Perceived baby size at birth (*n* = 7,423)			
Small	2,022	27.2	21.2[18.12,24.56]
Normal (average or above)	5,401	72.8	25.6[23.21,28.06]
Antenatal care visits (*n* = 7,473)			
No visit	2,804	37.5	10.2 [8.09,12.86]
1-4 visit	3,498	46.8	29.7 [26.74,32.89]
>4 visits	1,171	15.7	41.4 [37.15,45.69]
Place of delivery			
Home	5,171	69.1	8.8 [6.99,11.10]
Health facility	2,317	30.9	58.7 [54.98,62.24]
Cesarean section delivery			
No	7,353	98.2	24.0 [21.81,26.42]
Yes	135	1.8	35.6 [25.77,46.82]
Parity			
First birth	1,407	18.8	35.6 [31.01,40.50]
Second birth or beyond	6,081	81.2	21.6 [19.33,24.10]
Childbirth attendant			
Skilled attendant	2,431	32.4	56.9 [53.30,60.45]
Nonskilled attendant	4,011	53.6	8.0 [6.15,10.22]
No attendant	1,046	14.0	11.0 [7.51,15.77]

CI: confidence interval; EDHS: Ethiopian Demographic and Health survey; SNNP: Southern Nations, Nationalities, and People; SSC: skin-to-skin care.

**Table 2:** Factors affecting skin-to-skin care practice in Ethiopia, EDHS, 2016.

Explanatory variables	Practiced skin-to-skin care	
	COR [95% CI]	AOR [95% CI]
Mother's age		
< =18	1.00	1.00
19-35	1.08 [0.80, 1.46]	1.28 [0.87, 1.87]
> =35	0.67 [0.46, 0.99]	0.81 [0.47, 1.38]
Mother's education		
No formal education	1.00	1.00
Primary	1.92[1.60, 2.31]	1.03 [0.82, 1.29]
Secondary	5.31 [3.72, 7.57]	1.10 [0.68, 1.78]
Higher	4.51 [2.68, 7.60]	0.73 [0.36, 1.50]
Place of residence		
Urban	4.49 [3.34, 6.04]	1.27 [0.83, 1.93]
Rural	1.00	1.00
Region		
Tigray	1.00	1.00
Afar	0.21 [0.13, 0.32]	0.68 [0.43, 1.07]
Amhara	0.29 [0.20, 0.42]	0.57 [0.40, 0.82]^∗^
Oromia	0.28 [0.19, 0.42]	0.69 [0.44, 1.07]
Somali	0.19 [0.12, 0.29]	0.51 [0.31, 0.83]^∗^
Benishangul-Gumuz	0.47 [0.28, 0.78]	0.95 [0.62, 1.48]
SNNPR	0.34 [0.23, 0.50]	0.64 [0.43, 0.94]^∗^
Gambela	0.57 [0.37, 0.86]	0.71 [0.47, 1.08]
Harari	0.83 [0.53, 1.28]	1.00 [0.66, 1.49]
Addis Ababa	1.84 [1.21, 2.80]	0.94 [0.58, 1.54]
Dire Dawa	1.01 [0.66, 1.53]	1.05 [0.70, 1.57]
Wealth index quintile		
Poorest	1.00	1.00
Poorer	1.57 [1.12, 2.20]	1.10 [0.70, 1.72]
Middle	1.90 [1.35, 2.69]	1.28 [0. 86, 1.92]
Richer	2.37 [1.67, 3.37]	1.38 [0.89, 2.14]
Richest	5.88 [4.20, 8.23]	1.20 [0.72, 1.99]
Employment status		
Not employed	1.00	1.00
Employed	1.45 [1.20, 1.74]	1.02 [0.82, 1.27]
Marital status		
Never in union	1.00	1.00
Currently in union	0.49 [0.22, 1.11]	0.84 [0.30, 2.40]
Formerly in union	0.48 [0.20, 1.14]	0.80 [0.27, 2.39]
Sex of child		
Male	1.00	1.00
Female	1.17 [0.99, 1.39]	1.24 [1.01, 1.54]^∗^
Perceived baby size at birth		
Small	0.78 [.65, 0.94]	0.90 [0.71, 1.15]
Normal (average or above average)	1.00	1.00
Antenatal care visits		
No visits	1.00	1.00
1-4 visits	3.71 [2.80, 4.90]	1.51 [1.08, 2.12]^∗^
>4 visits	6.18 [4.61, 8.30]	1.43 [1.00, 2.05]
Place of delivery		
Home	1.00	1.00
Health facility	14.64 [11.13, 19.27]	4.51 [2.16, 9.44]^∗∗^
Cesarean section delivery		
No	1.00	1.0
Yes	1.75 [1.07, 2.84]	0.37 [0.23, 0.63]^∗∗^
Number of living children	0.87 [0.82,0.92]	1.05 [0.96, 1.16]
Parity		
First birth	2.01[1.59, 2.53]	1.14[0.81, 1.60]
Second birth or beyond	1.00	1.00
Childbirth attendant		
Skilled attendant	10.69 [6.83,16.73]	2.11 [0.90,4.94]
Nonskilled attendant	0.70 [0.47,1.05]	0.69 [0.46,1.05]
No attendant	1.00	1.00

^∗^Variables with *p* < 0.05 in the multivariable regression model, ^∗∗^variables with *p* < 0.01 in the multivariable regression model; AOR: adjusted odds ratio; CI: confidence interval; COR: crude odds ratio; SNNP: Southern Nations, Nationalities, and People; SSC: skin-to-skin care.

## Data Availability

The data were obtained from The DHS Program repository. The authors have no mandate to share the data set or make it publicly available. However, the data can be accessed upon a reasonable request from the DHS Program website (available at https://www.dhsprogram.com/data/available-datasets.cfm).

## Abbreviations

ANC: Antenatal care
AOR: Adjusted odds ratio
CI: Confidence interval
COR: Crudes odds ratio
CS: Cesarean section
DHS: Demographic and Health Survey
EAs: Enumeration areas
EDHS: Ethiopian Demographic and Health Survey
KMC: Kangaroo mother care
LBW: Low birth weight
SNNP: Southern Nations, Nationalities, and People's
SSC: Skin-to-skin care
WHO: World Health Organization.

## Supplementary Materials

Supplementary 1 Table S1: explanatory variables categorization and coding.

Supplementary 2 Table S2: STROBE 2007 (v4) Statement—checklist of items that should be included in reports of cross-sectional studies.

Supplementary 3 Table S3: the distribution of birth assistance by region and residence, EDHS 2016.
