# Supplementary material for: Maternal Health Service Uptake Is Associated with a Higher Skin-to-Skin Care Practice in Ethiopia: Result from a National Survey
Source: Biomed Res Int. 2020 Dec 16;2020:8841349. doi: 10.1155/2020/8841349 (PMC7768584; doi:10.1155/2020/8841349)
Supplement: Supplementary 1 — Table S1: explanatory variables categorization and coding. [file 8841349.f1.pdf]

Table S1: Explanatory variables categorization and coding

Variables	Category coding
Maternal age at delivery	1. <=18<br>2. 19-34<br>3. >=35
Place of residence	1. urban<br>2. rural
Regions	1. Tigray<br>2. Afar<br>3. Amhara<br>4. Oromia<br>5. Somali<br>6. Benishangul-Gumuz<br>7. SNNPR<br>8. Gambela<br>9. Harari<br>10. Addis Ababa<br>11. Dire Dawa
Antenatal care visits	0. No visit<br>1. 1-4 visits<br>2. >4 visits
Place of delivery	0. home<br>1. health facility

Wealth index quintile	1. poorest<br><br>2. poorer<br><br>3. middle<br><br>4. richer<br><br>5. richest
Maternal education	0. no formal education<br><br>1. primary<br><br>2. secondary<br><br>3. higher
Sex of child	1. male<br><br>2. female
Cesarean section delivery	0. No<br><br>1. Yes
Employment status	0. Not employed<br><br>1. Employed
Marital status	1. Never in union<br><br>2. Currently in union<br><br>3. Formerly in union
Perceived baby size at birth	1. Small<br><br>2. Normal (average or above average)
Parity	1. First birth<br><br>2. Second birth or above
Childbirth attendant	1. Non-skilled attendant

	2. Skilled attendant<br><br>3. No attendant
