# Supplementary material for: Maternal Health Service Uptake Is Associated with a Higher Skin-to-Skin Care Practice in Ethiopia: Result from a National Survey
Source: Biomed Res Int. 2020 Dec 16;2020:8841349. doi: 10.1155/2020/8841349 (PMC7768584; doi:10.1155/2020/8841349)
Supplement: Supplementary 3 — Table S3: the distribution of birth assistance by region and residence, EDHS 2016. [file 8841349.f3.pdf]

Table S3: The distribution of birth assistance by region and residence, EDHS 2016

**Variables**	**Birth attendant**						**Chi-square test**
	Skilled attendant		***Non-skilled attendant***		***No attendant***		
	Number	%	Number	%	Number	%	
**Residence**							
Urban	772	83.8	117	12.7	33	3.5	X^2=118.10; P $\leq$ 0.001
Rural	1,658	25.3	3,895	59.3	1,013	15.4	
**Region**							
Tigray	349	65.9	166	31.4	14	2.7	X^2=31.64; P $\leq$ 0.001
Afar	13	18.6	56	81.4	0	0	
Amhara	479	29.8	1,055	65.5	76	4.7	
Oromia	768	24.8	1,755	56.6	576	18.6	
Somali	57	21.5	203	76.5	6	2.1	
Benishangul-Gumuz	28	35.3	35	44.2	17	20.5	
SNNPR	524	33.0	710	44.8	351	22.2	
Gambela	10	49.4	8	40.3	2	10.30	

Harari	10	58.4	7	39.0	0.4	2.6
Addis Ababa	172	95.7	7	3.8	1	0.5
Dire Dawa	21	63.2	9	27.7	3	9.1
